# An Integrated Investigation of Atmospheric Gaseous Elemental Mercury Transport and Dispersion Around a Chlor-Alkali Plant in the Ossola Valley (Italian Central Alps)

**DOI:** 10.3390/toxics9070172

**Published:** 2021-07-18

**Authors:** Laura Fantozzi, Nicoletta Guerrieri, Giovanni Manca, Arianna Orrù, Laura Marziali

**Affiliations:** 1 Water Research Institute-National Research Council (IRSA-CNR), Largo Tonolli 50, I-28922 Verbania Pallanza, Italy; nicoletta.guerrieri@cnr.it (N.G.); arianna.orru@irsa.cnr.it (A.O.); 2 European Commission, Joint Research Centre (JRC), I-21027 Ispra, Italy; giovanni.manca@ec.europa.eu; 3 Water Research Institute-National Research Council (IRSA-CNR), Via del Mulino 19, I-20861 Brugherio, Italy; laura.marziali@irsa.cnr.it

**Keywords:** Ossola Valley, atmospheric gaseous elemental mercury, continuous Hg measurements, lichens, biomonitoring, mercury atmospheric sources

## Abstract

We present the first assessment of atmospheric pollution by mercury (Hg) in an industrialized area located in the Ossola Valley (Italian Central Alps), in close proximity to the Toce River. The study area suffers from a level of Hg contamination due to a Hg cell chlor-alkali plant operating from 1915 to the end of 2017. We measured gaseous elemental Hg (GEM) levels by means of a portable Hg analyzer during car surveys between autumn 2018 and summer 2020. Moreover, we assessed the long-term dispersion pattern of atmospheric Hg by analyzing the total Hg concentration in samples of lichens collected in the Ossola Valley. High values of GEM concentrations (1112 ng m^−3^) up to three orders of magnitude higher than the typical terrestrial background concentration in the northern hemisphere were measured in the proximity of the chlor-alkali plant. Hg concentrations in lichens ranged from 142 ng g^−1^ at sampling sites located north of the chlor-alkali plant to 624 ng g^−1^ in lichens collected south of the chlor-alkali plant. A north-south gradient of Hg accumulation in lichens along the Ossola Valley channel was observed, highlighting that the area located south of the chlor-alkali plant is more exposed to the dispersion of Hg emitted into the atmosphere from the industrial site. Long-term studies on Hg emission and dispersion in the Ossola Valley are needed to better assess potential impact on ecosystems and human health.

## 1. Introduction

Long-range atmospheric transport of mercury (Hg), its transformation to more toxic methylmercury (CH_3_Hg) and its bioaccumulation in the environment have motivated intensive research on the dynamics of Hg cycling over the last decades. As was recognized in 2013 by 127 world countries with the signing of the UNEP Minamata Convention, further scientific efforts are needed to improve atmospheric Hg monitoring and characterization of Hg sources in order to reduce risks associated with Hg contamination [1]. Hg is associated with global human health problems due to high toxicity, persistence and bioaccumulation in the aquatic food chain [2,3,4]. It is considered a pollutant of global concern and it is globally transported through the atmosphere as gaseous elemental mercury (GEM). GEM represents the prevalent form of Hg in the atmosphere (>95%) and is very stable in air, with a residence time of 0.5–2 years [5]. Other forms of atmospheric Hg are reactive gas phase Hg (RGM) and particulate phase Hg (TPM). RGM and TPM have a shorter atmospheric residence time than GEM (hours to days), and due to high water solubility and adsorption on particles, they tend to affect the terrestrial ecosystem locally or regionally by wet and dry deposition [6].

GEM evaporates easily and is emitted from sources of both natural and anthropogenic origin. Natural sources include volcanoes, geothermal activity, geological deposits of Hg minerals and re-emission of Hg previously deposited from air onto the surface of soil and water, whereas anthropogenic sources derive from mining (both for Hg and for other minerals) and extraction and burning of fossil fuels, which contain Hg as a trace contaminant (primarily coal), as well as production of metals, alkalis, cement and waste incineration [7,8]. Among industrial activities, GEM emission is prevalent especially at mining sites [9,10,11,12] and chlor-alkali plants using Hg-cell technology [13,14,15], but also in other Hg-related factories [16,17,18]. The atmosphere is the central pathway of dispersion for Hg emissions. From the atmosphere it can be deposited to earth surfaces, either by direct Hg^0^ dry deposition, or scavenging by wet and dry deposition after oxidation to RGM. Once deposited, land and ocean transformation processes play an important role in the cycling of Hg in terrestrial, freshwater and marine ecosystems, and in the production of more toxic CH_3_Hg compounds, which drives the most dangerous human exposure route through consumption of contaminated fish [19,20,21]. CH_3_Hg is a potent neurotoxin that can cause a variety of reproductive and developmental disorders at high concentrations [22]. New research has also suggested evidence that exposure to Hg may be a risk factor for cardiovascular disease [23]. Human exposure to Hg can occur through Hg vapor inhalation and less frequently through skin contact. Once in the body, Hg vapor is transported primarily to the brain, either dissolved in serum or adherent to red cell membranes, and has potential neurotoxic effects depending on the exposure dose [24].

The present research was focused on atmospheric Hg pollution in the Ossola Valley (Italian Central Alps), which hosts both natural (i.e., geogenic, due to the presence of cinnabar in the watershed) and anthropogenic sources of Hg. Among anthropogenic sources, a chlor-alkali plant operating from the First World War period to the end of 2017, is the main cause of Hg pollution in the study area [25,26]. The industrial discharges were released into the Toce River, which flows along the Ossola Valley, and were driven into Lake Maggiore, one of the main Italian lakes. Thus, contamination also reached the lake ecosystem, with strong implications on biota and, potentially, human health.

A large amount of data has been collected from 2000 to date on Hg contamination in water, sediments and biota of the Toce River and Lake Maggiore in the framework of the monitoring programs of the International Commission for the Protection of the Italian-Swiss Waters (CIPAIS; www.cipais.org; accessed on 15 March 2021) [25,27]. These data show that, even if the activity of the chlor-alkali plant was drastically reduced in the late 1990s, and finally stopped at the end of 2017, Hg contamination on the aquatic ecosystem did not show any significant reduction in sediments and biota in the last decade, confirming the high persistence of this toxicant and showing that active sources of the contaminant are still present, such as soil leaching and/or atmospheric deposition. Hg values in most fish species largely exceed the EU Environmental Quality Standard for biota [26,27,28]. 

To our knowledge, air Hg contamination in the Ossola Valley has not been studied before, even if the industrial area is a source of GEM because of the use of Hg-cell technology for the chlor-alkali process. From 2017, soil restoration has been in progress in the industrial area, and on-site soil treatment will be carried out. These operations may enhance Hg release from contaminated soil, with potential implications for human health of the resident population [29]. Worldwide recommendations for daily GEM chronic inhalation exposure report risk threshold values not to be exceeded ranging from 100 ng m^−3^ to 300 ng m^−3^ [30,31,32,33].

To conduct efficient monitoring programs in Hg contaminated areas, the integration of data obtained with different techniques is beneficial. Indeed, instrumental techniques, such as a laser radar system (LIDAR) [34], or compact instruments, such as Tekran, Gardis [35] and Lumex [36] analyzers, are excellent in measuring atmospheric Hg but represent only the average value during the sampling period and give information on Hg contamination at point sources. They have limited spatial resolution and limited applicability for estimating chronic population inhalation exposure and emissions.

Besides, many studies have used Hg bioaccumulation in lichens as a proxy for the average long-term Hg concentration in air, and to evaluate the spatial distribution of atmospheric Hg [37,38]. Many epiphytic lichens are sensitive to atmospheric pollutants and have been widely used for almost five decades as bioindicators of air quality and Hg pollution [39,40,41,42,43,44,45,46]. Their use is easy and cheap and allows location of hot spots of natural or anthropogenic emissions and assessment of spatiotemporal changes in Hg deposition patterns. As they receive nutrients directly from the atmosphere, lichens accumulate trace elements directly from the air, and concentrations of elements inside lichen thalli have been proved to correlate with their environmental levels [47]. Metal uptake can be both passive and active. Passive uptake is a physicochemical process influenced by metal ion concentrations, chemical status/speciation and climatic and environmental conditions, while active uptake is related to lichen metabolism and respiration rate [48]. Because of their slow growth rate, Hg concentration in lichens reflects the integrated value of Hg concentration in air over a time period, independently from weather conditions and fluctuations [45]. 

Comprehensive reviews on the application of moss and lichens in Hg biomonitoring, and on lichens as biological indicators of environmental quality, were published, respectively, by [37] and [38].

With the aim of assessing the impact of Hg pollution on air quality in the contaminated area around the chemical plant located along the Toce River, in the present study we investigated spatial and temporal changes in Hg distribution coupling short-term measurements of GEM concentrations in air to the measurement of Hg concentration in epiphytic lichens collected in the Ossola Valley at selected sampling points around the chemical plant.

Results were related to wind pattern along the Ossola Valley channel.

## 2. Materials and Methods

### 2.1. Study Area

**Figure 1:**
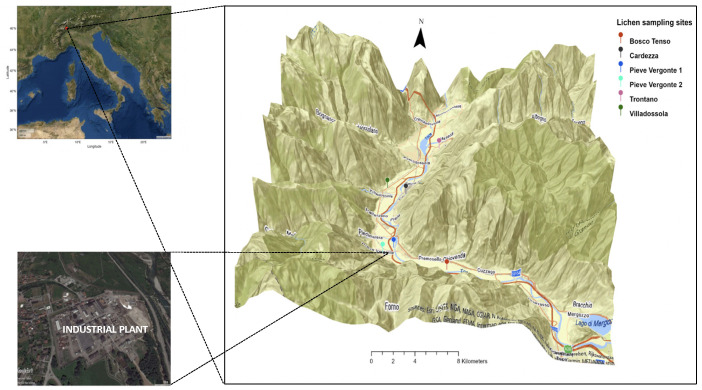
Location of the study area.

This research was carried out in the Ossola Valley (Italian Central Alps, Piedmont Region, Northern Italy) (Figure 1). The Ossola Valley is a large Alpine valley located on the Italian border with Switzerland. It comprises seven main side valleys and corresponds to a large part of the catchment area of the Toce River, which is one of the main tributaries of Lake Maggiore.

The valley hosts about 66,500 inhabitants and the main town is Domodossola, with about 18,000 inhabitants. Most industrial activities are localized close to this area. However, the biggest industrial plant is located close to Pieve Vergonte town (about 2493 inhabitants), and was used in the last century mainly for Dichlorodiphenyltrichloroethane (DDT), sulfuric acid and cloro-soda production. This latter product was obtained using a Hg-cell chlor-alkali plant, which was discontinued at the end of 2017 for conversion to non-Hg technology. The chlor-alkali facility reported total annual releases of Hg to air and water in the European Pollutant Release and Transfer Register (E-PRTR) managed by the European Environment Agency (https://prtr.eea.europa.eu/#/facilitydetails?FacilityID=162&ReportingYear=2017; accessed on 26 September 2020).

The climate of the Ossola Valley was defined through long-term (2002–2020) data collected at the Fomarco (hamlet of Pieve Vergonte) and Domodossola meteorological stations operated by Agenzia Regionale per la Protezione Ambientale del Piemonte (ARPA Piemonte, I-10035 Torino, Italy; http://www.arpa.piemonte.it/; accessed on 26 April 2021).

**Figure 2:**
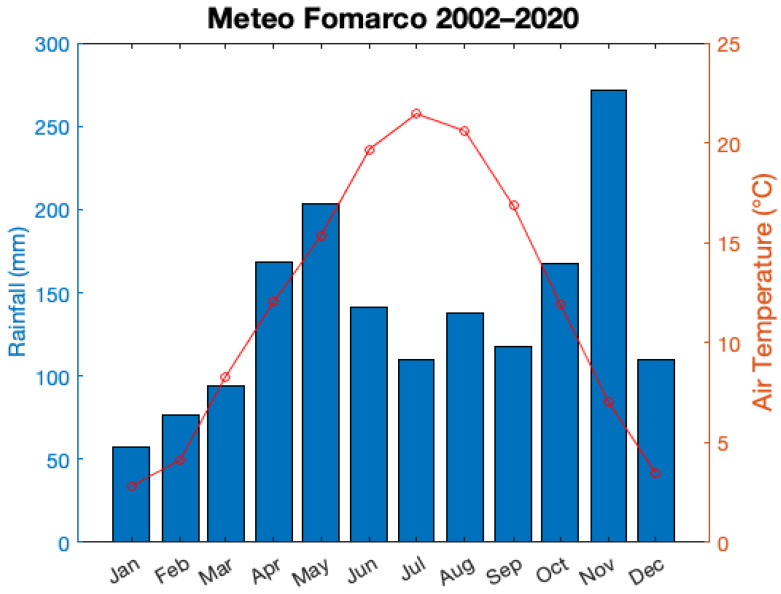
Mean monthly pattern of air temperature and total rainfall as recorded at the meteorological station in Fomarco (hamlet of Pieve Vergonte), operated by ARPA Piemonte (http://www.arpa.piemonte.it/; accessed on 26 April 2021). Air temperature values were calculated as averages of daily mean temperatures of a specific month over the period 2002–2020. Rainfall values represent averages of total monthly rainfall for a specific month over the period 2002–2020.

The mean annual temperature recorded at Fomarco is 12.5 °C, while average annual rainfall in Fomarco is 1366 mm. The seasonal trend of air temperature in the period 2002–2020 showed that July is the hottest month with an average temperature of 22.9 °C, while the coldest month is January with 1.9 °C (Figure 2). The valley has an alpine–sublitoral rainfall regime, that shows a bimodal pattern with two wet periods in autumn and spring, and two dry seasons, i.e., winter and summer (Figure 2).

**Figure 3:**
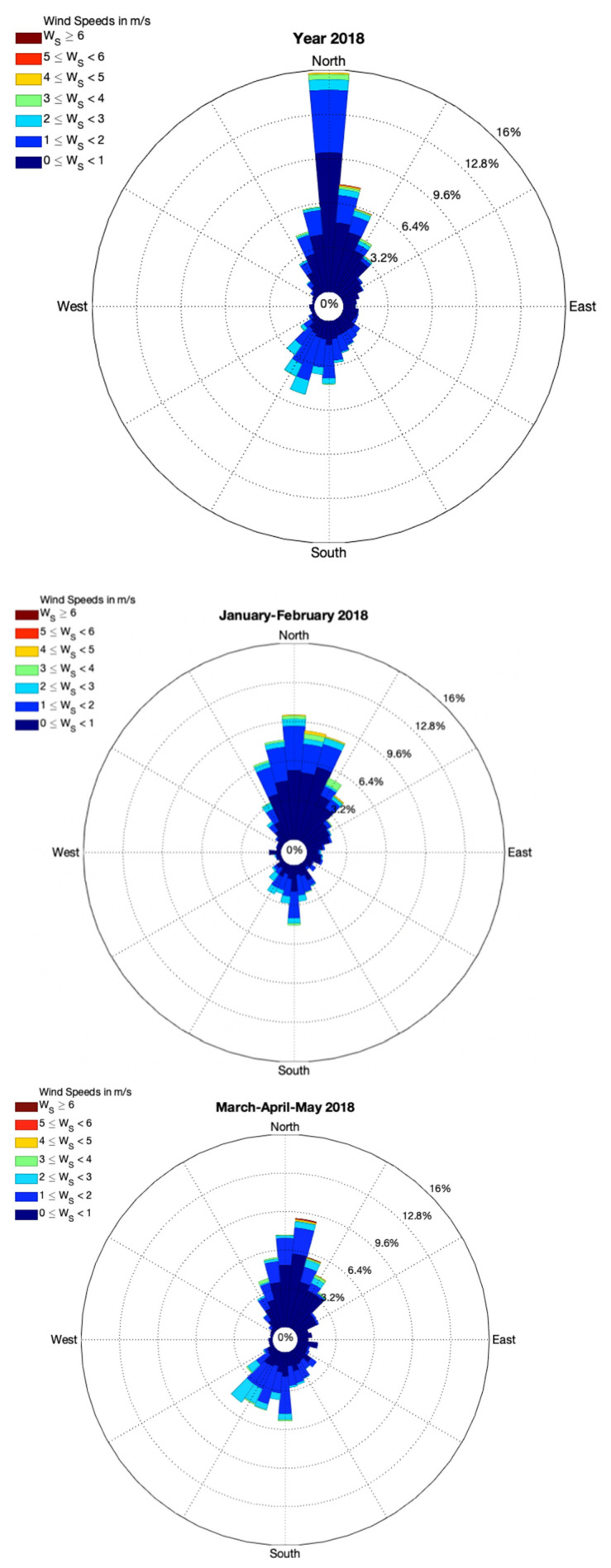

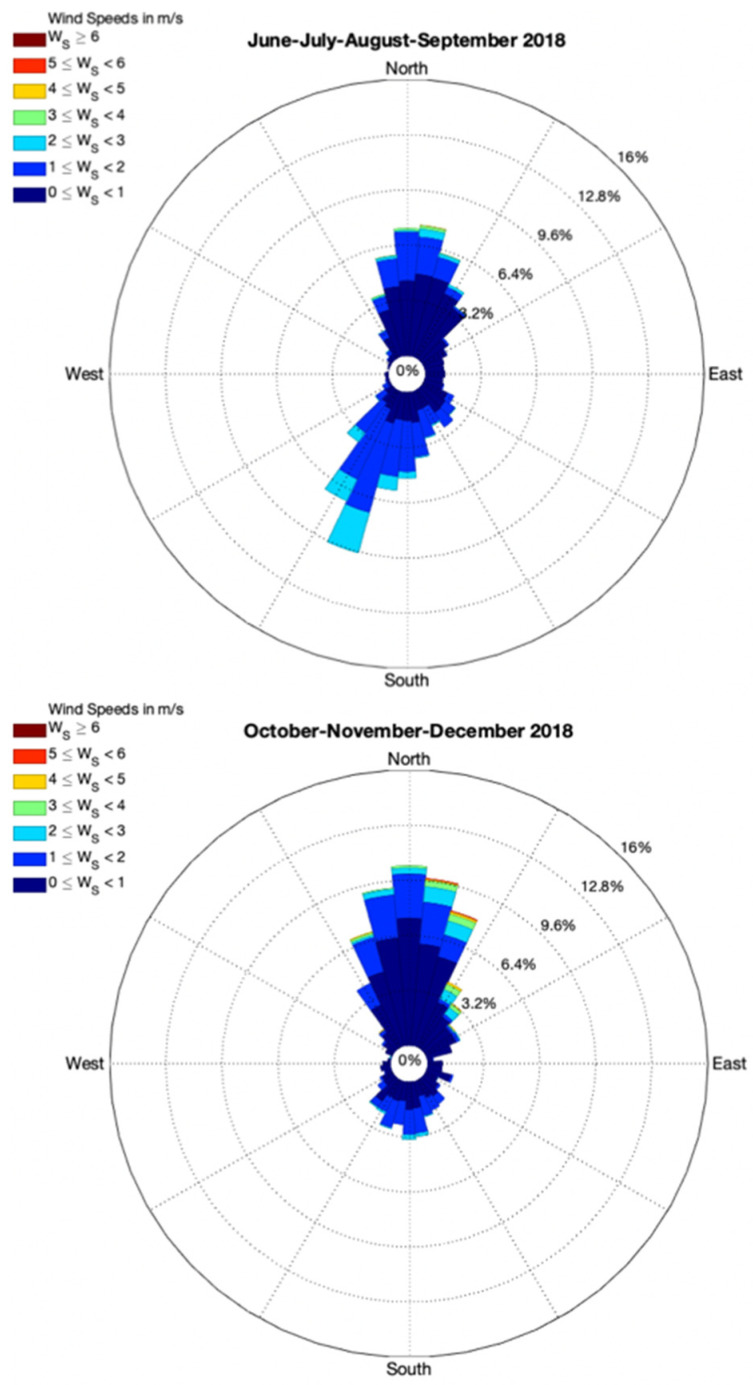
Yearly and seasonal wind roses from hourly values of wind direction and wind speed observed in 2018 at the Domodossola meteorological station operated by ARPA Piemonte (http://www.arpa.piemonte.it/; accessed on 26 April 2021).

The predominant wind on a yearly basis, as recorded at the Domodossola meteorological station of ARPA Piemonte, is from north sector. Figure 3 shows typical seasonal and yearly wind regimes as observed in Domodossola. Winds are driven by local forcing, and thus mountain and valley winds are very common when synoptic forcing is small or missing. This occurs especially in summer, when temperature gradients are higher than in winter. In this case, winds are from the north sector during the night and from south-southwest during the day. Synoptic winds are mainly from North.

### 2.2. Determination of GEM in Air 

Atmospheric GEM concentration was measured between autumn 2018 and summer 2020. Measurements were made by using a portable time-saving instrument model RA915+ (Lumex, Saint Petersburg, Russia), which is able to produce a large number of reliable data, highly comparable with those produced by CVAFAS (Cold Vapor Atomic Fluorescence Spectrometry). This instrument has a high sensitivity and selectivity and is able to provide real time measurement with a response time of 1 s, being the only Hg analyzer not requiring gold amalgam preconcentration and subsequent regeneration steps. The instrument function is based on differential atomic absorption spectrometry using high frequency modulation of light polarization (ZAAS-HFM) [49]. The effect of interfering impurities is avoided due to the application of a Zeeman background correction system. The detection limit (DL) is governed by shot noise and equals DL = 2 ng m^−3^ (average time being 1 s) and DL = 0.3 ng m^−3^ (average time being 30 s) for Hg determination in air. The dynamic range covers four orders of magnitude (2–30,000 ng m^−3^) [49]. The accuracy of the method is 20% [50]. GEM measurements were carried out with a response time of 10 s during car surveys carried out from a moving car or from a fixed location. Air was sampled at 1.5 m height above the ground. Data acquisition was made by using RAPID Software by Lumex.

Geographic location of single data was assigned through a GPS provided by a commercial mobile phone.

### 2.3. Lichen Collection and Preparation for Analysis

Epiphytic lichens of the species *Parmelia tiliacea* were collected from the beginning of March to the beginning of May 2019.

The lichen *P. tiliacea* was previously used for its ability to accumulate metals, its broad distribution and easy identification [51,52]. It is a widely distributed species in south-western Europe, one of the most common in the Mediterranean basin and the most common lichen species in the study area. It has the advantage of easy separation from the bark, being a foliose lichen with big leaves, and is easily identifiable by morphology [51,53].

Lichen species were identified in the field.

Lichen sampling sites (Trontano, Villadossola, Cardezza, Pieve Vergonte 1, Pieve Vergonte 2, Bosco Tenso) are located in the riparian zone along the Toce River (Figure 1). A large part of the study area is in a “lichen desert” zone because it is heavily anthropized and characterized by the presence of private homes and agricultural crops.

Lichen samples were collected along a north-south transect with respect to the position of the Pieve Vergonte chlor-alkali plant, starting from Trontano (13 km north from the industrial area) to Bosco Tenso (1 km South-East), in both the industrial area and surrounding areas.

The southeast sampling site (BoscoTenso), once a World Wide Fund for nature (WWF) site (0.22 km^2^ extension), is located close to the town of Premosello-Chiovenda, and is one of the few wooded area in the study area; vegetation is dominated by lime trees and oaks.

In each sampling site, six lichen subsamples were collected from trunks of standing trees of three different trees at 1.5–2.0 m from the ground to avoid soil contamination [42]. The circumference of the trees was >60 cm.

The collected lichens were placed in Falcon tubes, stored in a cooler box and transported to the laboratory. All samples were cleaned from extraneous matter, washed with deionized water by immersion for few seconds, air-dried at room temperature and then freeze-dried (72 h at −45 °C and 0.1 atm).

### 2.4. Analytical Procedure for the Determination of Hg in Lichens

Lichens were homogenized using a Retsch MM2000 ball mill (Retsch Technology GmbH, Haan, Germany). Total Hg concentrations in lichens were determined by thermal decomposition and amalgamation and atomic absorption spectrometry according to US-EPA method 7473 [54] using an Automated Mercury Analyzer (AMA-254, FKV Srl, Bergamo, Italy). The instrument detection limit was 0.01 ng Hg and the working range was 0.05 to 600 ng Hg. The limit of quantification (LOQ) calculated as ten times the standard deviation of the blank and considering a sample mass (plant tissue) of 25 mg, was 0.009 mg kg^−1^.

For accuracy evaluation, the certified reference material BCR-CRM061 aquatic moss powder (Community Bureau of Reference, Institute for Reference Materials and Measurements, Geel, Belgium) was analyzed (reference value = 0.23 ± 0.02 mg kg^−1^), obtaining a mean recovery of 103.8 ± 1.1% (*n* = 3) of certified values. Precision was checked by triplicate analysis, and the coefficient of variation was ≤5%.

## 3. Results

**Figure 4:**
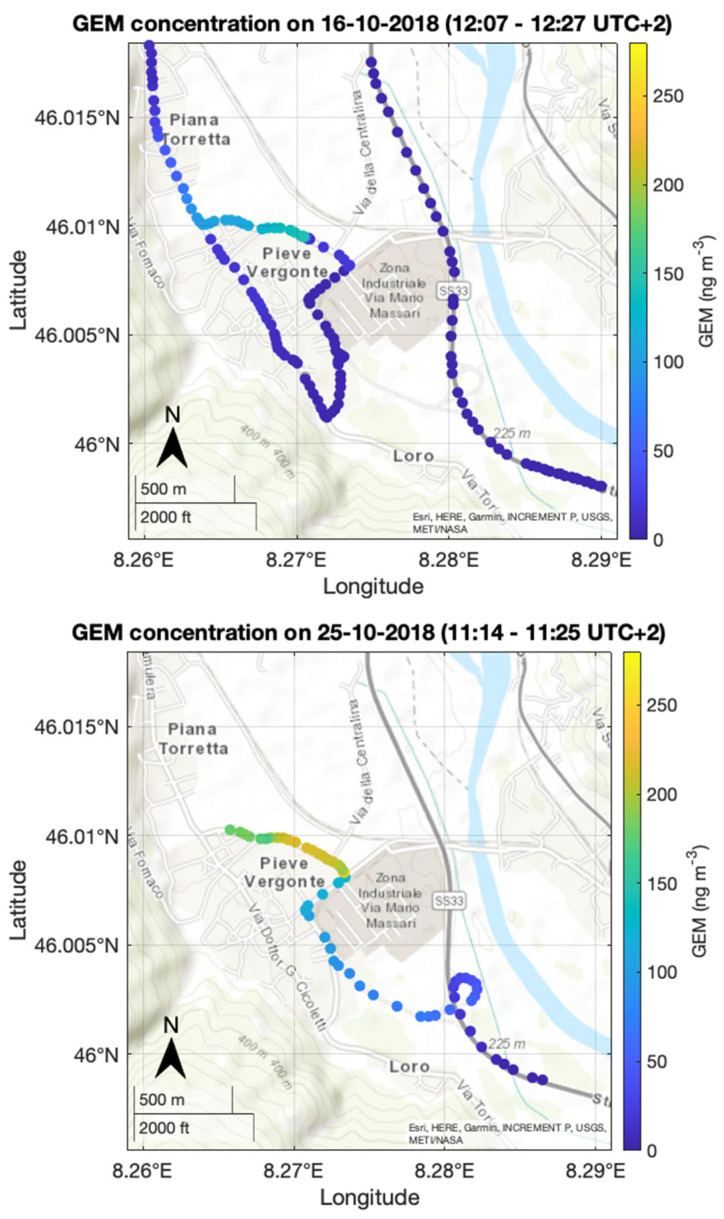

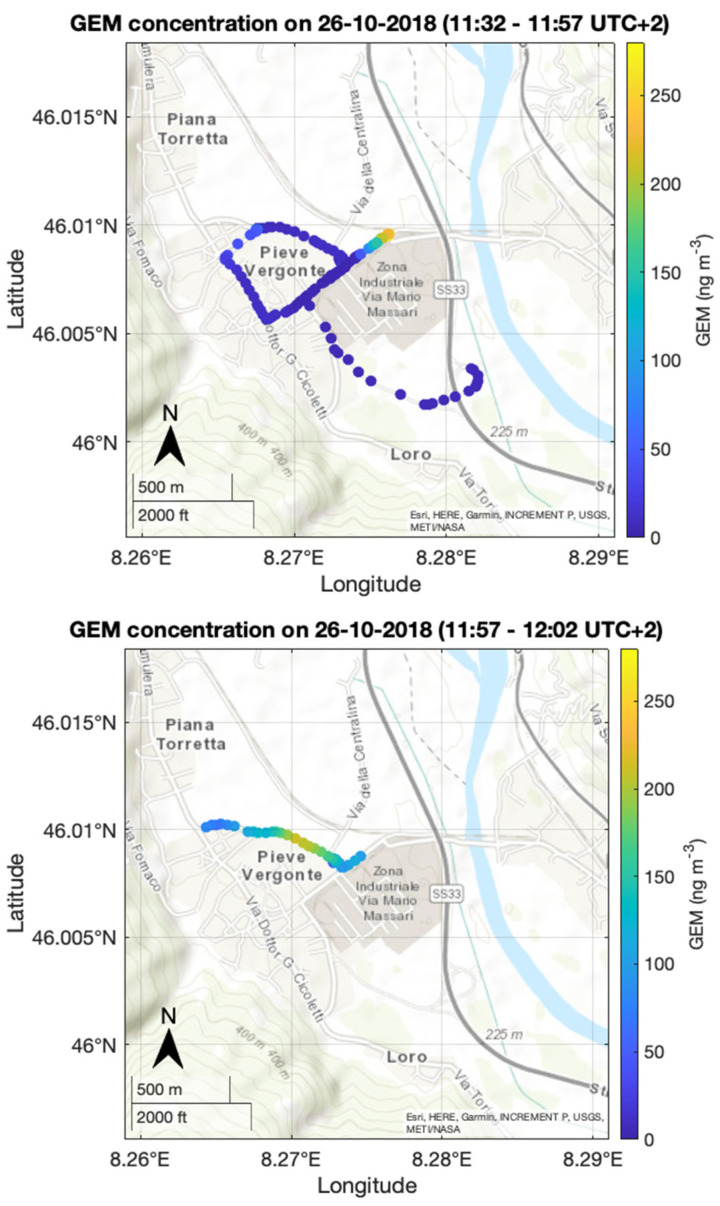
GEM concentrations measured during four car surveys around the industrial area in Pieve Vergonte.

Spatial distribution of GEM concentration of some selected car surveys carried out between autumn 2018 and summer 2020 around the chlor-alkali plant located near the Toce River is showed in Figure 4.

GEM values recorded during the car survey on 16 October 2018 were in the range 1–152 ng m^−3^, with maximum values observed near the chlor-alkali plant. The following car surveys on 25 and 26 October showed even higher GEM values near the industrial area. Indeed, the highest GEM concentration on 25 October was 267 ng m^−3^, and maximum values observed on October 26th during the car surveys before and after noon were 280 ng m^−3^ and 258 ng m^−3^, respectively. Hourly mean air temperature measured in the period of car surveys, showed in Figure 4, was in the range 16–20 °C with an hourly wind direction between 112° and 231°. Hourly averages of wind speed were in the range 0.6–0.9 m s^−1^.

**Figure 5:**
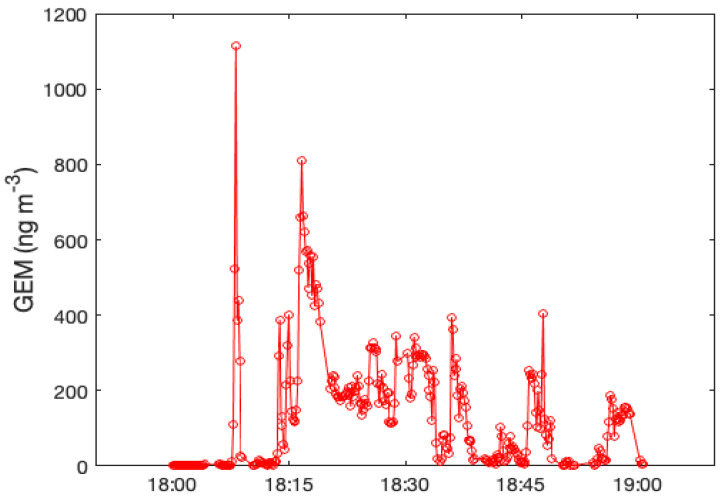
Temporal variation of GEM in a time span of 1 h measured on the 28th of June 2020 in proximity of the chlor-alkali plant of Pieve Vergonte.

On 28 June 2020, we left the Lumex RA-915+ recording GEM concentrations continuously for one hour (from 18:00 to 19:00 local time, UTC + 2) near the main entrance of the industrial area. This short time series showed high variability of GEM with a maximum reaching the value of 1112 ng m^−3^ (Figure 5). During this sampling, air temperature was 22.9 °C, wind speed was 0.7 m s^−1^ and wind direction was 200°.

These results highlight that GEM concentrations in air were often higher than the typical terrestrial background concentration in the Northern Hemisphere (~1.5–1.7 ng m^−3^) [19]. Moreover, GEM concentrations observed in close proximity of the chlor-alkali plant suggest the presence of a direct source of mercury, despite the fact that the plant had been closed since the end of 2017. Background values of the valley (~2 ng m^−3^) were reached within a radius of 2 km from the plant. However, as can be seen in Figure 4, a Hg plume heading from the chemical plant towards the town of Pieve Vergonte was observed. 

**Figure 6:**
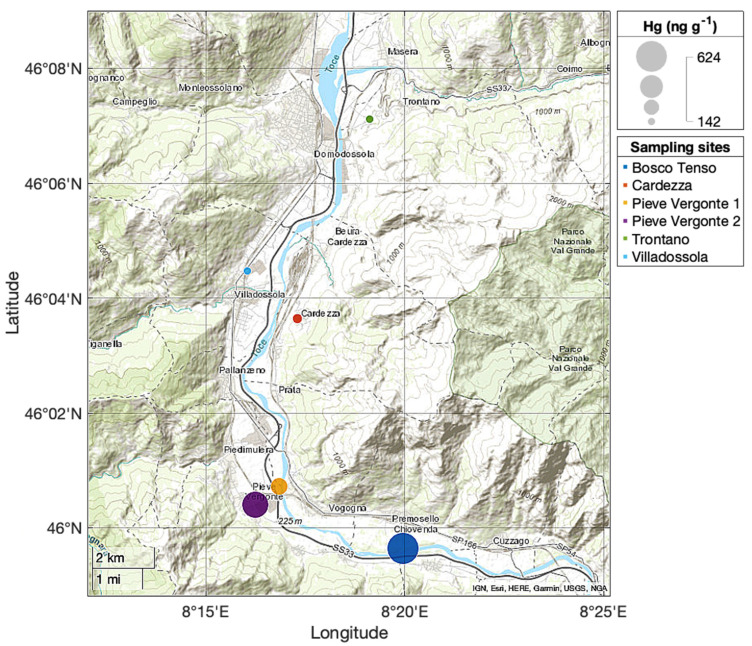
Total Hg concentrations (ng g^−1^ d.w.) in lichens of the species *P. tiliacea* collected in five sampling sites along a North-South transect in the Ossola Valley in March–May 2019.

The analytical results for Hg in the lichen *P. tiliacea* collected at the six sites around the chlor-alkali plant are summarized in Table 1 and reported in Figure 6. Total Hg concentrations range from 142 ng g^−1^ to 624 ng g^−1^. 

Total Hg concentrations in samples of *P. tiliacea* collected north of the industrial area showed values significantly lower than those collected in the vicinity of the industrial area. Values were close to background Hg concentrations measured in lichens collected from nonpolluted areas of the northern hemisphere (Table 2).

An increasing trend in lichen concentrations was observed from north to south along the valley channel (Figure 6). The highest Hg concentration (624 ng g^−1^ d.w) was observed in samples of *P. tiliacea* collected at the most southern sampling site (Bosco Tenso).

The typical wind pattern in the Ossola Valley (prevailing Northerly winds, Figure 3) fosters dispersion of Hg emitted by the chlor-alkali plant along the valley channel in areas south of the industrial area.

## 4. Discussion

**Figure 7:**
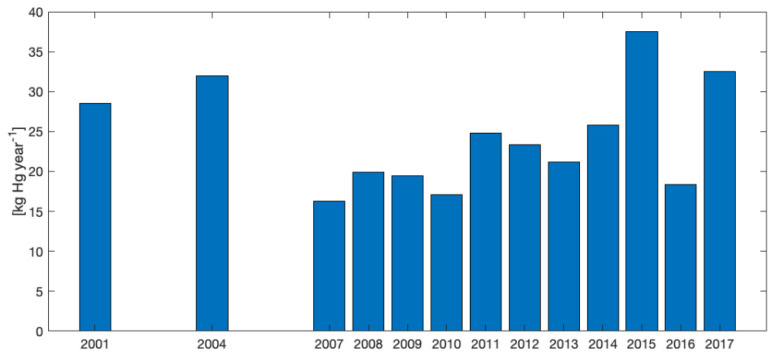
Total annual release of Hg to air as reported in the E-PRTR database by the industrial facility located in Pieve Vergonte.

GEM concentrations observed in the present study demonstrated the presence of a local source of Hg contamination related to past industrial activities. We recorded the highest GEM concentrations (up to 1112 ng m^−3^) in close proximity to the chlor-alkali plant located near the Toce River in the Ossola Valley. Several studies have reported the presence of a long history of Hg pollution for the aquatic ecosystem in the study area due to the presence of this plant [25,26,27,28,59,60,61]. Hg emissions into the atmosphere reported by the chlor-alkali facility in the E-PRTR database range from 16 kg Hg y^−1^ to 37 kg Hg y^−1^ in the period 2001–2017 (Figure 7).

Reported emissions to air demonstrated that the chlor-alkali plant was still active until 2017, acting as a point source of mercury for the atmosphere. Moreover, the same facility reported in the E-PRTR database for the same period a total annual release of Hg to the aquatic environment ranging from 1.05 to 4.60 kg Hg y^−1^. Not surprisingly, mercury values in sediments and biota in the Toce River downstream of the industrial site were significantly higher than those measured upstream [25], and no decreasing trends were observed in 2008–2018 period [28].

GEM concentrations measured in proximity of the chlor-alkali plant were up to three orders of magnitude higher than background values in the northern hemisphere. However, concentrations observed during car surveys highlighted the presence of mercury-polluted air masses moving from the chlor-alkali plant towards the town of Pieve Vergonte (Figure 4). As GEM measurements were performed in the late morning, when the atmosphere is well mixed, nighttime concentrations could be even higher because the bottom portion of the atmospheric boundary layer is characterized by statically stable air with weaker, sporadic turbulence [36,62]. The Minimal Risk Level (MRL) for daily GEM chronic inhalation exposure reported by the U.S. Agency for Toxic Substances and Disease Registry (ATSDR, 2015) is 200 ng m^−3^. The U.S. Environmental Protection Agency (EPA) indicates a reference value for inhalation of GEM of 300 ng m^−3^ [32], while the World Health Organization [30] and the International Programme on Chemical Safety [31] report even lower values of 100–200 ng m^−3^. This first survey highlights the presence of a potential exposure pathway driven by wind direction, which is still active even after the discontinuance of the factory. This exposure is more pronounced during spring and summer seasons because in this period there is a higher frequency of southerly winds along the valley channel (Figure 3).

The analysis of Hg concentration in lichens allowed us to examine with a complementary approach the impact of airborne Hg contamination in the surrounding areas close to the chemical plant. Lichen sampling was begun about 1 year after production at the chlor-alkali plant was discontinued, but we are confident that Hg values observed in lichens still represent air quality conditions in place before the discontinuance. In fact, the authors of [62] also found that a significant decrease in elemental content would be expected within a year or two from the reduction in emission of an industrial source. The authors of [63] developed a mathematical model involving a simple representation of the lichen and a memory loss function, and concluded that the time needed to reach a new equilibrium with changed bioavailability of airborne elements is specific for each element and depends on ambient and lichen morphophysiological conditions. The authors of [64] estimated a reduction of about one order of magnitude in Hg concentrations and a Hg residence half-life of 1.6 years with a mean life of 2.3 years in two species of epiphytic lichens collected in the vicinity of a chlor-alkali plant after five years from a slowdown of industrial activity and the introduction of more stringent environmental controls. 

Measured values in the most upstream stations (142–159 ng g^−1^) were close to background Hg concentrations measured in lichens collected from nonpolluted areas of the northern hemisphere (Table 2), while values at the sites close to the industrial area (261–624 ng g^−1^) were in line with values measured in other sites contaminated by chlor-alkali plant activity (Table 3).

A clear north-south gradient of Hg uptake in lichens along the valley channel, with the highest Hg concentration measured in samples of *P. tiliacea* collected at the southern sampling point (Bosco Tenso), was highlighted (Figure 6).

A number of literature studies report a direct correlation of Hg concentration in lichens with the distance from Hg emission source. One study [41] found Hg concentrations decreasing from 2510 to 70 ng g^−1^ at increasing distances from a chlor-alkali plant located near Grenoble, with a contamination radius of about 2 km, while [40] measured a background value of Hg (148 ng g^−1^) in epiphytic lichens collected at a distance of 8 km from a chlor-alkali plant in Canada and the highest Hg concentration (3660 ng g^−1^) at 250 m distance. A concentration decreases within a radius of 3 km in lichens collected at increasing distances from abandoned mining and smelting plants (Abbadia San Salvadore, Italy) was observed by [37] and [67]. A comparative study of Hg concentrations in air and in epiphytic lichens in a former Hg mining area in Idrija, Slovenia, was performed by [39], who found a good correlation between Hg concentrations in air and lichens. 

In our study we observed a specific spatial gradient of Hg in lichens along the valley channel, and the highest concentration of total Hg was not observed near the industrial area (261–474 ng g^−1^) but at the Bosco Tenso site (624 ng g^−1^; 4.5 km away from the chlor-alkali plant). The spatial gradient of Hg contamination can be explained considering the topography of the Ossola Valley. In fact, our finding is in agreement with literature data showing that in industrial areas where metallic Hg is handled, prevailing wind direction and topography are accountable for the dispersion pattern in the surrounding areas [14]. These authors used Hg concentration in lichens to construct a dispersion model for the atmospheric Hg dispersion around a chlor-alkali plant in the Flix area (NE Spain) on the basis of topographical data and meteorological parameters, and did not find correlations between lichen Hg contents and distance to the chlor-alkali plant. In [62], a very weak correlation between lichen Hg contents and distance to the main source of an ex-chlor-alkali plant located in Torviscosa (Northern Italy) was found, and it was concluded that the dispersion of GEM was driven by the prevalent wind direction, thus confirming that lichens are a useful tool for long-term airborne Hg biomonitoring. 

The Ossola Valley has a complex geomorphology. It is deeply incised in the bedrock and is characterized by the hydrographic basin of the meandering Toce River and by mountain peaks sometimes exceeding 3000 m a.s.l. The upper valley, where northern lichen sampling sites were located (Trontano, Cardezza, Villadossola), is wider with respect to the lower valley at the southern lichen sampling site (Bosco Tenso) surrounded by 1000–1500 m high mountains. The peculiar morphology of the Ossola Valley plays a fundamental role in modifying the atmospheric boundary-layer flow that undergoes a channeling along the valley, determining the observed spatial pattern of lichen contamination. In addition, it is necessary to take into account that another factor may have influenced the higher concentrations of total Hg measured in lichens collected at the Bosco Tenso site. In fact, in wooded areas the uptake capacity of lichen is higher than in open areas [68]. Moreover, in these areas the Hg deposition occurs mostly through throughfall and litterfall rather than through atmospheric precipitation [69]. As a consequence, it must be highlighted that an increase in ambient air levels of Hg results in an increase of Hg burden in terrestrial and aquatic ecosystems, possibly leading to elevated concentrations of methylmercury in trophic chains. Such a situation might have an important bearing on acceptable levels of mercury in the atmosphere.

## 5. Conclusions

Data presented in this paper provide the first evidence of atmospheric GEM concentration and its deposition in areas surrounding a chlor-alkali plant located near the Toce River in the Ossola Valley (Italian Central Alps).

Determining or predicting Hg concentration in the atmosphere is of crucial importance in identifying sensitive areas requiring ecosystem and health protection. We showed how an integrated investigation of airborne Hg analytical measurements and biomonitoring can be employed to locate Hg emission sources and to identify areas where Hg inhalation exposure may be of concern.

Our study highlights that the wind pattern in the Ossola Valley and its morphology imply that the area located south of the chlor-alkali plant is more affected by dispersion of Hg emitted into the atmosphere from the industrial site. The ongoing reclamation at Pieve Vergonte industrial site will likely decrease the mercury emissions, but contaminated soils around the facility will remain a long-term potential source. Therefore, further studies are necessary to avoid or minimize risks for ecosystems and human health deriving from the dispersion of this airborne Hg. In this sense, greater effort should be directed towards continuous GEM measurements, together with an in-depth study of how meteorological factors (air temperature, soil temperature, solar radiation, wind speed and rainfall) could help understand Hg emission/deposition cycles, Hg dispersion and potential future contamination trends. Moreover, a better characterization of soil chemico-physical properties should be planned, together with measurements of Hg emission from soil, to improve our knowledge on Hg cycling and soil potential for Hg volatilization.

## Figures and Tables

**Table 1:** Total Hg concentration in lichens samples. ^a^ Sites coordinates of lichens; ^b^ Mean ± sd (ng g^−1^, dry weight); ^c^ Distance from the center of the chlor-alkali plant (0.3 km radius).

Sites	Coordinates ^a^	Total Hg (ng g^−1^) ^b^	km ^c^	Elevation (m a.s.l.)
Trontano	46°07′06.79′′ N 08°19′07.39′′ E	142 ± 20	10.0	270
Villadossola	46°04′28.08′′ N 08°16′02.83′′ E	142 ± 5	7.5	255
Cardezza	46°03′38.40′′ N 08°17′18.23′′ E	159 ± 7	6.0	420
Pieve Vergonte 1	46°00′23.72′′ N 08°16′14.76′′ E	474 ± 13	0.3	232
Pieve Vergonte 2	46°00′43.08′′ N 08°16′50.23′′ E	261 ± 4	0.7	220
Bosco Tenso	45°59′38.59′′ N 08°19′57.47′′ E	624 ± 13	4.5	210

**Table 2:** Background Hg concentration in lichens collected from clean areas around the world (from [45], modified).

Country	Mean ± SD (ng g^−1^)	References
USA, Alaska	43 ± 4.3	[55]
USA, Yellowstone Park	140 ± 50	[56]
Slovenia	110 ± 10	[39]
Finland	223 ± 76	[57]
France, Grenoble	70 ± 7.0	[41]
Italy, Tuscany	170 ± 80	[58]
South Africa, Limpopo	60 ± 8.0	[45]

**Table 3:** Total Hg concentrations (ng g^−1^) measured in lichens sampled in different sites contaminated by chlor-alkali plant industrial activity. * Mean ± sd.

Country	Total Hg Concentrations (ng g^−1^)	References
Torviscosa (ITA)	80–380	[65]
Rosignano Solvay (ITA)	740 ± 46 *	[66]
Flix (ESP)	40–3700	[14]
Grenoble (FRA)	70–2510	[41]
New Brunswick (CAN)	148–3660	[40]

## Data Availability

All supporting data have been included in this study and are available from the corresponding authors upon request.
